# Auswirkungen der COVID-19-Pandemie auf die stationäre Dermatochirurgie in Deutschland: Retrospektive Auswertung der operativen Fälle aus neun Hautkliniken

**DOI:** 10.1007/s00105-024-05417-5

**Published:** 2024-10-10

**Authors:** Galina Balakirski, Chalid Assaf, Edgar Dippel, Anne Fröhlich, Lukas Kofler, Alexander Kreuter, Christian Kunte, Daniela Hartmann, Silke C. Hofmann, Thomas Horn, Thorsten Neubert, Teodora Pumnea, Laurenz Schmitt, Amir S. Yazdi, Christoph R. Löser

**Affiliations:** 1 Zentrum für Dermatologie, Allergologie und Dermatochirurgie, Helios Universitätsklinikum Wuppertal, Universität Witten/Herdecke, Heusnerstr. 40, 42283 Wuppertal, Deutschland; 2 Klinik für Dermatologie und Venerologie, Helios Klinikum Krefeld, Krefeld, Deutschland; 3 Hautklinik, Klinikum Ludwigshafen am Rhein, Ludwigshafen, Deutschland; 4 Zentrum für Hauterkrankungen, Universitätsklinikum Bonn, Bonn, Deutschland; 5 Hautklinik, Universitätsklinikum Tübingen, Tübingen, Deutschland; 6 Hautzentrum am Holzmarkt, Holzmarkt 6, 88400 Biberach, Deutschland; 7 Klinik für Dermatologie, Venerologie und Allergologie, Helios St. Elisabeth Klinik Oberhausen, Oberhausen, Deutschland; 8 Klinik für Dermatologie, Venerologie und Allergologie, Helios St. Johannes Klinik Duisburg, Duisburg, Deutschland; 9 Abteilung für Dermatochirurgie und Dermatologie, Artemed Fachklinik München, München, Deutschland; 10 Klinik und Poliklinik für Dermatologie und Allergologie, LMU München, München, Deutschland; 11 Klinik für Dermatologie und Allergologie, Universitätsklinikum der RWTH Aachen, Aachen, Deutschland; 12 Hautklinik, Universitätsklinikum Heidelberg, Heidelberg, Deutschland

**Keywords:** Demografische Daten, Malignes Melanom, Langzeitwirkungen, Dermatochirurgisches Erkrankungsspektrum, Versorgungslage, Demographic analysis, Malignant melanoma, Long-term effect, Dermatosurgical spectrum of disease, Care situation

## Abstract

**Einleitung:**

Aktuell existieren nur wenige Daten über die Beeinträchtigung der stationären dermatochirurgischen Versorgung in deutschen Hautkliniken durch die COVID-19-Pandemie.

**Methoden:**

Es erfolgte eine retrospektive Auswertung aller dermatochirurgischen Fälle, die in den Jahren 2019, 2020 und 2021 in 9 deutschen Hautkliniken in 4 Bundesländern stationär behandelt wurden. Die Diagnosen wurden anhand der ICD-10-Codes erfasst. Zusätzlich wurden demografische Daten wie Alter und Geschlecht sowie die stationäre Verweildauer ausgewertet.

**Ergebnisse:**

In den Jahren 2019, 2020 und 2021 wurden jeweils 10.739, 9185 und 9828 dermatochirurgische Patienten stationär behandelt. Somit betrug die Reduktion der stationären dermatochirurgischen Fälle im Jahr 2020 14,5 % und im Jahr 2021 8,5 % im Vergleich zum Jahr 2019. Die stationäre operative Versorgung der Melanome ging im Jahr 2020 um 10,1 % der Fälle zurück. Im Jahr 2021 betrug dieser Rückgang nur noch 1,4 % im Vergleich zum Jahr 2019. Die stationäre operative Versorgung benigner Veränderungen wie Melanozytennävi oder Viruswarzen zeigte in beiden Pandemiejahren eine starke Regression.

**Diskussion:**

Unsere Daten erfassen erstmalig und repräsentativ die Entwicklung der stationären Versorgung des gesamten dermatochirurgischen Erkrankungsspektrums im Rahmen der COVID-19-Pandemie in Deutschland. Nach dem initial starken Rückgang der stationären dermatochirurgischen Fälle im Jahr 2020 zeigte sich im Jahr 2021 eine geringere Differenz zu 2019. Diese Tendenz kann als Hinweis gedeutet werden, dass für eine stationäre dermatochirurgische Versorgung weiterhin ein starker Bedarf besteht, der bis dato ambulant nicht aufgefangen werden kann.

**Zusatzmaterial online:**

Die Online-Version dieses Artikels (10.1007/s00105-024-05417-5) enthält 3 zusätzliche Tabellen. Beitrag und Zusatzmaterial stehen Ihnen im elektronischen Volltextarchiv auf https://www.springermedizin.de/die-dermatologie zur Verfügung.

## Abstract (en)

**Introduction:**

Currently, only little data is available on the impact of the COVID-19 pandemic on inpatient dermatosurgical care in German dermatological clinics.

**Methods:**

A retrospective analysis of all dermatosurgical cases that were treated in inpatient setting in nine German dermatological clinics in four federal states in 2019, 2020 and 2021 was performed. The diagnoses were recorded using the ICD-10 codes. In addition, demographic data such as age, gender and the length of inpatient stay were analysed.

**Results:**

In 2019, 2020 and 2021, a total of 10,739, 9185 and 9828 dermatosurgical inpatients were treated respectively. Thus, the reduction of inpatient dermatosurgical cases was 14.5% in 2020 and 8.5% in 2021 compared to 2019. Inpatient surgical treatment of melanoma decreased by 10.1% of cases in 2020. This decrease was only 1.4% in 2021 compared to 2019. The number of inpatient surgeries performed for benign lesions such as melanocytic nevi or viral warts reduced sharply in both pandemic years.

**Conclusion:**

Our data show for the first time how inpatient care for the entire spectrum of dermatosurgical diseases developed during the COVID-19 pandemic in Germany. After the initial marked decline in inpatient dermatosurgical cases in 2020, there was less difference in 2021 compared to 2019. This trend can be interpreted as an indication that there is still a strong need for inpatient dermatosurgical care that cannot yet be met on an outpatient basis.

Im Frühling 2023, fast 3 Jahre seit dem Ausbruch des SARS-CoV-2-Virus in Deutschland, wurden aufgrund der stabilen epidemiologischen Lage alle noch verbliebenen Corona-Schutzmaßnahmen aufgehoben, und es wurde offiziell das Ende der COVID-19-Pandemie erklärt [1, 2]. Auch wenn die medizinische Versorgung in Praxen und Krankenhäusern aktuell wieder ohne Einschränkungen funktioniert, ist es wichtig, die Beeinträchtigungen der Patientenversorgung während der COVID-19-Pandemie zu erfassen, um epidemiologische Entwicklungen besser zu verstehen, Rückschlüsse auf die aktuelle Versorgungslage zu ziehen und möglichen Langzeitwirkungen der Pandemie entgegenzuwirken.

Als integraler Bestandteil der Dermatologie umfasst die Dermatochirurgie verschiedene Aufgaben [3]. Hierzu zählen die operative Therapie bösartiger Hauttumoren, die Behandlung der Hidradenitis suppurativa, die operative Versorgung gutartiger Läsionen, die Behandlung von chronischen Wunden, ästhetische Eingriffe und vieles mehr [3]. Der negative Einfluss der COVID-19-Pandemie auf die dermatochirurgische Versorgung wurde bereits in anderen europäischen Ländern (wie Italien, Großbritannien und Frankreich) und den USA untersucht. So ist insbesondere während des Lockdowns die Anzahl der in den Kliniken durchgeführten Eingriffe stark gesunken, teilweise um mehr als die Hälfte im Vergleich zum gleichen Zeitraum vor der Pandemie [4–6]. Daraus resultierte eine Verzögerung in der Diagnostik und Therapie der malignen Tumoren. Dies konnte beispielsweise bei den während der COVID-19-Pandemie diagnostizierten malignen Melanomen festgestellt werden, welche eine Zunahme der Tumordicke und höherer Tumorstadien aufwiesen [7–10]. Viele Autoren geben allerdings zu bedenken, dass die bislang vorliegenden Daten möglicherweise nicht das gesamte Ausmaß des Einflusses der SARS-CoV-2-Pandemie auf die Dermatologie und Dermatochirurgie wiedergeben und erwarten auch nach Beendigung der Pandemie weitere Auswirkungen auf die Gesundheitskosten, die Morbidität und die Überlebensrate von Patienten mit Hauttumoren [11, 12].

Bis dato liegen nur vereinzelt Daten über den Einfluss der COVID-19-Pandemie auf die Dermatochirurgie in Deutschland vor. So berichteten im Rahmen einer bundesweiten Umfrage 58 % der deutschen dermatologischen Kliniken, zu Beginn der Pandemie viele elektive Eingriffe absagen bzw. verschieben zu müssen. Ein Großteil der befragten Kliniken berichtete jedoch, dass notwendige Eingriffe, beispielsweise bei malignem Melanom oder anderen großen oder schnell wachsenden Tumoren, weiterhin durchgeführt werden konnten [13]. Eine retrospektive bundesweite Auswertung der Versicherungsdaten zeigte einen Rückgang der in deutschen Hautkliniken stationär operierten malignen Melanome um 7 % und der epithelialen Tumoren („non-melanoma skin cancer“ [NMSC]) um 16 % im Zeitraum vom 18.03.2020 bis 17.03.2021 im Vergleich zum Jahr zuvor [14]. Diese Zahlen stehen im Einklang zur kürzlich festgestellten Reduktion der an das Bayerische Krebsregister gemeldeten Melanomfälle um über 10 % im Zeitraum von März 2020 bis Februar 2021 im Vergleich zum gleichen Zeitraum vor dem Ausbruch der COVID-19-Pandemie [15]. Auch wenn in einzelnen dermatologischen Kliniken in Nordrhein-Westfalen die Anzahl der neu diagnostizierten und operativ versorgten malignen Melanome und Plattenepithelkarzinome in den Jahren 2020 und 2021 nicht gegenüber dem Jahr 2019 reduziert war, zeigte sich dennoch eine gewisse diagnostische bzw. therapeutische Verzögerung. So wurde in den beiden Pandemiejahren eine signifikante Zunahme der positiven Schildwächterlymphknoten bei malignen Melanomen im Vergleich zum Jahr 2019 beobachtet [16]. Bei Plattenepithelkarzinomen wurde eine signifikante Zunahme der besonders dicken Tumoren mit einer Tumordicke über 6 mm in den Jahren 2020 und 2021 im Vergleich zum Jahr 2019 festgestellt [17].

Um die Auswirkungen der COVID-19-Pandemie auf das gesamte Spektrum der stationären Dermatochirurgie in Deutschland besser zu beschreiben, wurde die vorliegende, multizentrische Studie initiiert.

## Methoden

### Patientendaten

Es wurden retrospektiv die Daten aller dermatochirurgischen Patienten ausgewertet, die an den teilnehmenden Zentren im Zeitraum vom 01.01.2019 (1 Jahr vor der COVID-19-Pandemie) bis zum 31.12.2021 (Jahr des Beginns der Pandemie und 1 Jahr nach dem Beginn der Pandemie) stationär aufgenommen wurden.

Die Diagnosen wurden anhand des ICD-10-Codes (ICD-10-GM; Internationale Klassifikation der Krankheiten[International Statistical Classification of Diseases and Related Health Problems] mit deutscher Anpassung [German Modification]), erfasst und das Procedere wurde mittels Operationen- und Prozedurenschlüssel (OPS) geprüft, um festzustellen, ob es sich um eine primär dermatochirurgische stationäre Aufnahme gehandelt hat.

Folgende Parameter wurden ausgewertet: die nach ICD-10 codierte Hauptdiagnose, das Patientenalter, das Geschlecht sowie die stationäre Verweildauer und der Zeitpunkt der stationären Aufnahme (Monat, Quartal, Jahr).

Patienten mit konservativen dermatologischen Diagnosen, die lediglich eine diagnostische Intervention (wie beispielsweise eine Hautbiopsie) gemäß den codierten OPS-Ziffern erhalten haben, wurden ausgeschlossen. Auch Patienten, die ausschließlich ein Wunddébridement (beispielsweise bei Ulcus cruris) gemäß den codierten OPS-Ziffern und nachfolgend ausschließlich eine konservative Therapie der Erkrankung erhalten haben, wurden ebenfalls ausgeschlossen. Patienten mit Ulcus cruris, die gemäß den codierten OPS-Ziffern eine Wunddeckung (beispielsweise eine Spalthauttransplantation) erhalten haben, wurden jedoch in die Auswertung eingeschlossen.

Für alle teilnehmenden Zentren lag ein positives Ethikvotum der jeweils zuständigen Ethikkommission vor oder eine Bestätigung der zuständigen Ethikkommission, dass für diese Studie mit retrospektiver Datenauswertung kein formales Votum notwendig sei.

Insgesamt nahmen an der Auswertung 9 deutsche dermatologische Kliniken mit einem Schwerpunkt in der Dermatochirurgie teil (vgl. Online-Tab. 1).

### Statistische Auswertung

Um die Auswertung der Daten übersichtlich zu gestalten, wurden ähnliche Diagnosen zu Diagnosegruppen zusammengefasst (Online-Tab. 2 und 3). Die Datenauswertung erfolgte überwiegend deskriptiv. Für die nominalskalierten Variablen (wie Diagnosen und Geschlecht der Patienten) wurden Häufigkeitstabellen erstellt, und die Signifikanz der Veränderungen im Laufe der Zeit wurde mittels Chi-Quadrat-Test (bei Signifikanzniveau von 5 %) berechnet. Für die metrischen Daten (dargestellt als Mittelwert ± Standardabweichung) wurde die Signifikanz der Veränderungen im Laufe der Zeit mittels ANOVA für unabhängige Stichproben (Signifikanzniveau von 5 %) bestimmt. Zusätzlich wurde die logistische Regression zur Erfassung der Korrelationen zwischen den nominalskalierten Variablen angewendet. Die statistische Auswertung wurde mittels der Programme SPSS (Version 22.0, SPSS, Inc., Chicago, IL, USA) und Microsoft Excel 2007 für Windows durchgeführt und visualisiert.

## Ergebnisse

### Allgemeine und demografische Daten

**Abb. 1:**
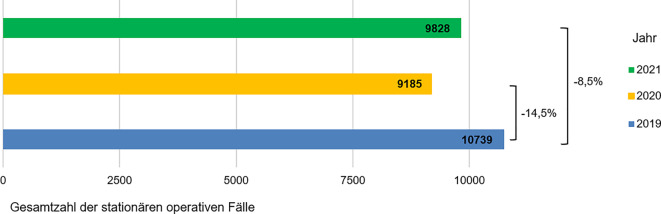
Darstellung der Entwicklung der dermatochirurgischen stationären Fälle in den Jahren 2020 und 2021 in den dermatologischen Abteilungen unabhängig von der Hauptdiagnose mit prozentualem Rückgang der stationären operativen Patienten im Vergleich zum Jahr 2019 (angenommen als 100 %)

In den Jahren 2019, 2020 und 2021 wurden an den teilnehmenden Kliniken jeweils 10.739, 9185 und 9828 dermatochirurgische Patienten stationär behandelt (Tab. 1; Abb. 1). Das mittlere Patientenalter in den ersten beiden Pandemiejahren (66,9 ± 21,8 Jahre in 2020 und 66,9 ± 22,4 Jahre in 2021) war ähnlich zu dem in 2019 (66,4 ± 22,6 Jahre) (*p* = 0,121; Tab. 2). Auch die Geschlechtsverteilung in den Jahren 2020 und 2021 zeigte keinen statistisch signifikanten Unterschied zum Jahr 2019 (Tab. 1). Allerdings wurden in den Jahren 2020 und 2021 statistisch signifikant weniger Kinder und Jugendliche (< 18 Jahren) stationär operiert als im Vergleich zum Jahr 2019. Der Anteil der stationär operierten Kinder und Jugendlichen betrug in 2019 1,5 % aller stationären dermatochirurgischen Patienten (162/10.739) verglichen mit 1,0 % (90/9185) in 2020 und 0,7 % (66/9828) in 2021 (*p* < 0,001). Die Verteilung der stationären Fälle zwischen den Universitätskliniken und nichtuniversitären Kliniken veränderte sich im Verlauf der 3 Jahre nicht (*p* = 0,444; Tab. 1).

**Tab. 1:** Geschlechts- und Altersverteilung der in den Jahren 2019 bis 2021 stationär behandelten dermatochirurgischen Patienten in den dermatologischen Abteilungen

	Jahr			*p*-Wert
	2019	2020	2021	
Anzahl der stationären Fälle insgesamt	10.739	9185	9828	*<0,001*
Stationäre Fälle aus Universitätskliniken (%)	5376 (50,1 %)	4679 (50,9 %)	4942 (50,3 %)	0,444
Stationäre Fälle aus Nicht-Universitätskliniken (%)	5363 (49,9 %)	4506 (49,1 %)	4886 (49,7 %)	
Anzahl männlicher Patienten (%)	6120 (57,0 %)	5200 (56,6 %)	5533 (56,3 %)	0,606
Anzahl weiblicher Patienten (%)	4619 (43,0 %)	3985 (43,4 %)	4295 (43,7 %)	
Anzahl der Patienten im Alter von ≥18 Jahre (%)	10.577 (98,5 %)	9095 (99,0 %)	9762 (99,3 %)	*<0,001*
Anzahl der Patienten <18 Jahre (%)	162 (1,5 %)	90 (1,0 %)	66 (0,7 %)

**Tab. 2:** Mittleres Alter und stationäre Verweildauer der stationären dermatochirurgischen Patienten, die in den Jahren 2019 bis 2021 in den dermatologischen Abteilungen behandelt wurden

	Jahr			*p*-Wert
	2019	2020	2021	
	Mittelwert ± SD	Mittelwert ± SD	Mittelwert ± SD	
Alter der Patienten (in Jahren)	66,38 ± 22,65	66,91 ± 21,82	66,95 ± 22,38	0,121
Stationäre Verweildauer (in Tagen)	3,95 ± 4,05	3,64 ± 3,37	3,71 ± 3,96	*<0,0001*

*SD* Standardabweichung

Die mittlere stationäre Verweildauer verkürzte sich von 3,9 ± 4,0 Tagen im Jahr 2019 auf 3,6 ± 3,4 bzw. 3,7 ± 4,0 Tage in jeweils 2020 und 2021, was sich als statistisch signifikant erwies (< 0,0001; Tab. 2).

**Abb. 2:**
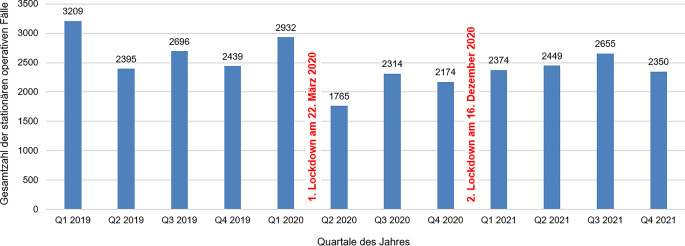
Entwicklung der stationären operativen Fälle in den dermatologischen Abteilungen in den Jahren 2019 bis 2021, aufgeschlüsselt nach den Quartalen des jeweiligen Jahres. Der stärkste Einbruch der stationären dermatochirurgischen Patientenzahlen kann nach Beginn des 1. Lockdowns im 2. Quartal 2020 beobachtet werden

Betrachtet man die Entwicklung der stationären dermatochirurgischen Zahlen quartalsweise, so kann ein stabiles Verteilungsmuster mit einem starken Einbruch der stationären Aufnahmen im 2. Quartal 2020 erkannt werden (Abb. 2).

### Stationäre Dermatochirurgie maligner Tumoren

**Abb. 3:**
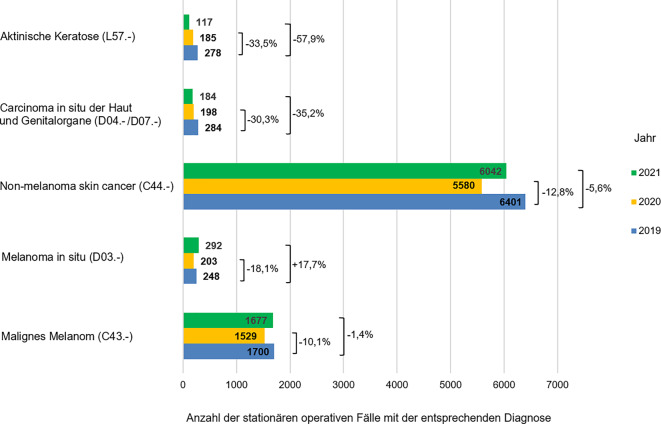
Darstellung der stationär operierten malignen Melanome und epithelialen Tumoren („non-melanoma skin cancer“) sowie In-situ-Läsionen in den Jahren 2019 bis 2021 mit prozentualem Rückgang dieser Diagnosen in den Jahren 2020 und 2021 im Vergleich zum Jahr 2019 (als 100 % angenommen)

In allen 3 untersuchten Jahren stellten epitheliale Hauttumoren („non-melanoma skin cancer“, ICD-Code 44.‑) die häufigste stationäre dermatochirurgische Diagnose dar (Tab. 3). Dennoch kam es im Jahr 2020 zu einer Reduktion dieser Diagnose um 12,8 % (821 Fälle) im Vergleich zum Jahr vor Beginn der COVID-19-Pandemie. Im Jahr 2021 kam es zu einem leichten Anstieg der unter stationären Bedingungen operierten epithelialen Hauttumoren, sodass die Reduktion der entsprechenden stationären Fälle im Vergleich zum Jahr 2019 nur 5,6 % (359 Fälle) betrug (Abb. 3). Die zweithäufigste stationäre dermatochirurgische Diagnose stellte das maligne Melanom dar. Im 1. Pandemiejahr kam es zum Rückgang der stationär operierten malignen Melanome um 10,1 % (171 Fälle) im Vergleich zum Jahr 2019. Im Jahr 2021 war dagegen die Anzahl der stationär operierten malignen Melanome verglichen zum Jahr vor Beginn der COVID-19-Pandemie um nur 1,4 % (27 Fälle) gesunken (Abb. 3).

**Tab. 3:** Darstellung der absoluten Zahlen aller Diagnosen, die in den Jahren 2019 bis 2021 stationär operativ in den dermatologischen Abteilungen behandelt wurden

Diagnosen	Jahr		
	2019	2020	2021
Anogenitale (venerische) Warzen	21	29	17
Viruswarzen	78	52	33
Molluscum contagiosum	26	15	23
Bösartige Neubildungen der Lippen, Zunge oder Gingiva	58	56	53
Malignes Melanom	1700	1529	1677
Epitheliale Hauttumoren („non-melanoma skin cancer“)	6401	5580	6042
Bösartige Neubildung des Bindegewebes (u. a. Sarkome)	121	111	129
Bösartige Neubildung des Genitoanalbereichs	11	11	9
Sekundäre bösartige Neubildung jeder Lokalisation	95	79	91
(Kutane) Lymphome	86	57	69
Melanoma in situ	248	203	292
Carcinoma in situ der Haut und Genitalorgane	284	198	184
Aktinische Keratose	278	185	117
Gutartige Neubildung des Bindegewebes	151	119	125
Melanozytennävus	183	112	126
Gutartige Neubildungen der Haut und sonstiger Lokalisationen	130	131	141
Neubildung unsicheren oder unbekannten Verhaltens (u. a. atypisches Fibroxanthom)	54	59	63
Chronisches Ulkus der Haut (inklusive Ulcus cruris) unabhängig von der Genese	48	60	44
Sonstige Krankheiten des Anus und des Rektums	80	40	44
Pilonidalzyste	32	30	32
Krankheiten der Nägel	29	12	18
Rhinophym	30	35	27
Follikuläre Zysten der Haut und der Unterhaut (beispielsweise Atherom oder Trichilemmalzyste)	56	49	64
Hidradenitis suppurativa	193	161	180
Hautabszess, Furunkel, Karbunkel oder Phlegmone	78	65	47
Sonstige Krankheiten der männlichen Genitalorgane	82	55	53
Sonstige Krankheiten der weiblichen Genitalorgane	14	10	4
Sonstige angeborene Fehlbildungen der Haut und Gefäße	24	39	27
Hyperhidrose	8	4	7
Komplikationen bei Eingriffen	75	58	53
Sonstiges	65	41	37
*Gesamt*	*10.739*	*9185*	*9828*

Sowohl für die Diagnose Carcinoma in situ (ICD-Code D04.-/D07.‑) als auch für aktinische Keratosen (L57.‑) zeigte sich ein noch deutlicherer Rückgang der stationären dermatochirurgischen Fälle und betrug im Jahr 2020 jeweils 30,3 % (86 Fälle) und 33,5 % (93 Fälle) im Vergleich zu 2019 (Abb. 3). Obwohl die Anzahl der stationären Falle mit der Diagnose malignes Melanom (ICD-Code C43.‑) oder bösartige Neubildung der Haut (ICD-Code C44.‑) im Jahr 2021 gegenüber dem Jahr 2020 wieder leicht anstieg, war für die Diagnose Carcinoma in situ und aktinische Keratose kein Anstieg zu verzeichnen. Hier kam es vielmehr zu einer weiteren Reduktion der stationären Fälle um jeweils 35,2 % (100 Fälle) und 57,9 % (161 Fälle) im Vergleich zum Jahr 2019. Bei der Diagnose Melanoma in situ (ICD-Diagnose D03.‑) zeigte sich im Jahr 2020 zunächst eine Reduktion der stationären Fälle um 18,1 % (48 Fälle) verglichen mit dem Jahr 2019, in 2021 kam es jedoch zu einer Zunahme um 17,7 % (44 Fälle).

**Abb. 4:**
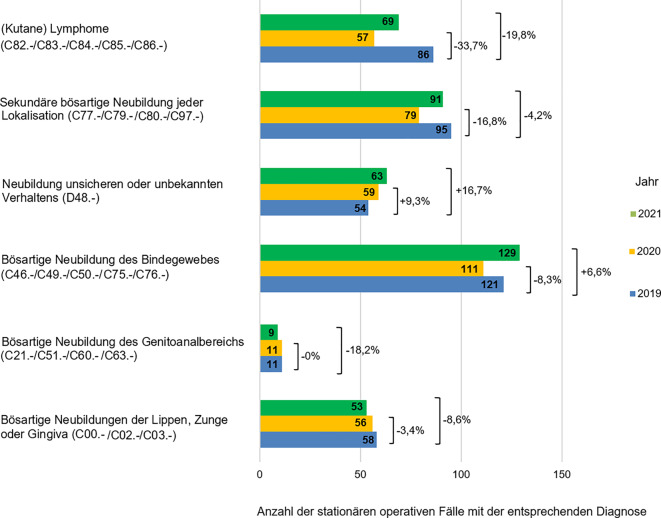
Darstellung der Fallzahlen der stationär operierten bösartigen Hautveränderungen in den Jahren 2019 bis 2021 mit prozentualem Rückgang der häufigsten Diagnosen in den Jahren 2020 und 2021 im Vergleich zum Jahr 2019 (als 100 % angenommen)

Es zeigte sich auch eine leichte Reduktion der operativ versorgten kutanen Lymphome im Vergleich zu 86 Patienten im Jahr 2019 um 33,7 % (29 Fälle) und 19,8 % (17 Fälle) in jeweils 2020 und 2021. Dagegen zeigte sich eine leichte Zunahme der Fallzahlen bei der Diagnose Neubildungen unsicheren oder unbekannten Verhaltens (ICD-Diagnose D48.-, unter anderem atypisches Fibroxanthom, ICD-Diagnose D48.5) um jeweils 9,3 % (5 Fälle) und 16,7 % (9 Fälle) im Jahr 2020 bzw. 2021 im Vergleich zum Jahr vor der COVID-19-Pandemie. Die bösartigen Neubildungen des Bindegewebes (ICD-Diagnosen C46.-, C49.-, C50.-, C75.-, C76.-, unter anderem Sarkome der Haut) wiesen im Jahr 2020 eine Fallzahlreduktion um 8,3 % (10 Fälle) auf im Vergleich zum Jahr 2019. Im Jahr 2021 zeigte sich jedoch eine Zunahme der stationären dermatochirurgischen Fälle mit dieser Diagnose um 6,6 % (9 Fälle) (Abb. 4). Veränderungen der stationären Zahlen der weiteren, seltenen malignen Tumoren in den beiden Pandemiejahren im Vergleich zum Jahr 2019 sind in der Abb. 4 dargestellt.

### Stationäre Dermatochirurgie benigner Hautläsionen

**Abb. 5:**
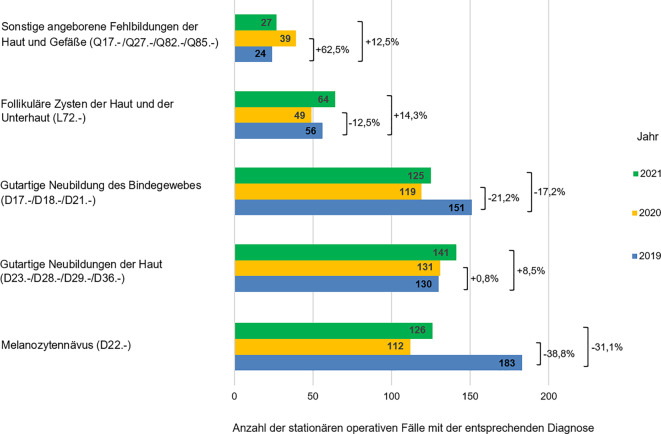
Darstellung der stationär operierten gutartigen Hautläsionen in den Jahren 2019 bis 2021 mit prozentualem Rückgang der häufigsten Diagnosen in den Jahren 2020 und 2021 im Vergleich zum Jahr 2019 (als 100 % angenommen)

Die Anzahl der stationär operierten melanozytären Nävi (ICD-Diagnose D22.‑) sank im Jahr 2020 um 38,8 % (71 Fälle) im Vergleich zum Vorjahr. Diese Tendenz setzte sich im Jahr 2021 fort (Abb. 5). Auch die Anzahl der stationär operierten gutartigen Neubildungen des Bindegewebes (ICD-Diagnosen D17.-, D18.- und D21.‑) sank im Jahr 2020 und 2021 um jeweils 21,2 % (32 Fälle) und 17,2 % (26 Fälle) im Vergleich zum Jahr 2019. Im Gegensatz dazu zeigte sich für die Diagnosen gutartige Neubildungen der Haut (ICD-Diagnosen D23.-, D28.-, D29.- und D36.‑) und sonstige angeborene Fehlbildungen der Haut und Gefäße (ICD-Diagnosen Q17.-, Q27.-, Q82.- und Q85.‑) eine Zunahme der stationären dermatochirurgischen Fälle während der beiden Pandemiejahre im Vergleich zum Jahr 2019 (Abb. 5).

### Stationäre operative Versorgung weiterer Erkrankungen

**Abb. 6:**
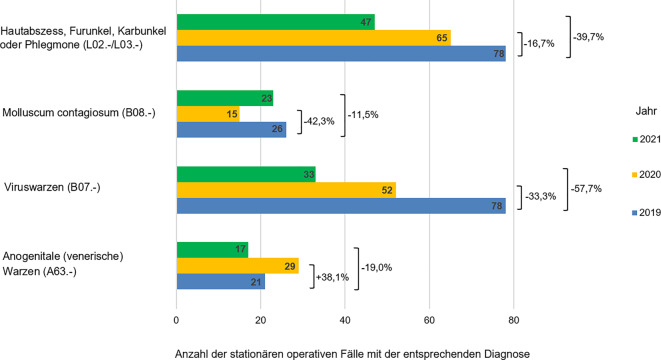
Darstellung der stationär operierten Hautläsionen infektiöser Genese in den Jahren 2019 bis 2021 mit prozentualem Rückgang der häufigsten Diagnosen in den Jahren 2020 und 2021 im Vergleich zum Jahr 2019 (als 100 % angenommen)

Die Anzahl weiterer dermatologischer Krankheitsbilder, die im stationären Rahmen operativ versorgt wurden, stellte sich sehr inhomogen dar. So zeigte sich eine deutliche Reduktion der stationären Fälle der Viruswarzen (ICD-Diagnose B07.‑) sowohl im Jahr 2020 (um 33,5 % [26 Fälle]) als auch im Jahr 2021 (um 57,7 % [45 Fälle]) im Vergleich zum Jahr 2019. Auch zeigte sich ein Rückgang der stationär operierten Hautabszesse, Furunkel und Karbunkel (ICD-Diagnosen L02.- und L03.‑) (Abb. 6). Dennoch ergab sich ein Zuwachs der stationär operierten Condylomata acuminata (ICD-Diagnose A63.‑) im Jahr 2020 um 38,1 % (8 Fälle) im Vergleich zum Jahr 2019.

**Abb. 7:**
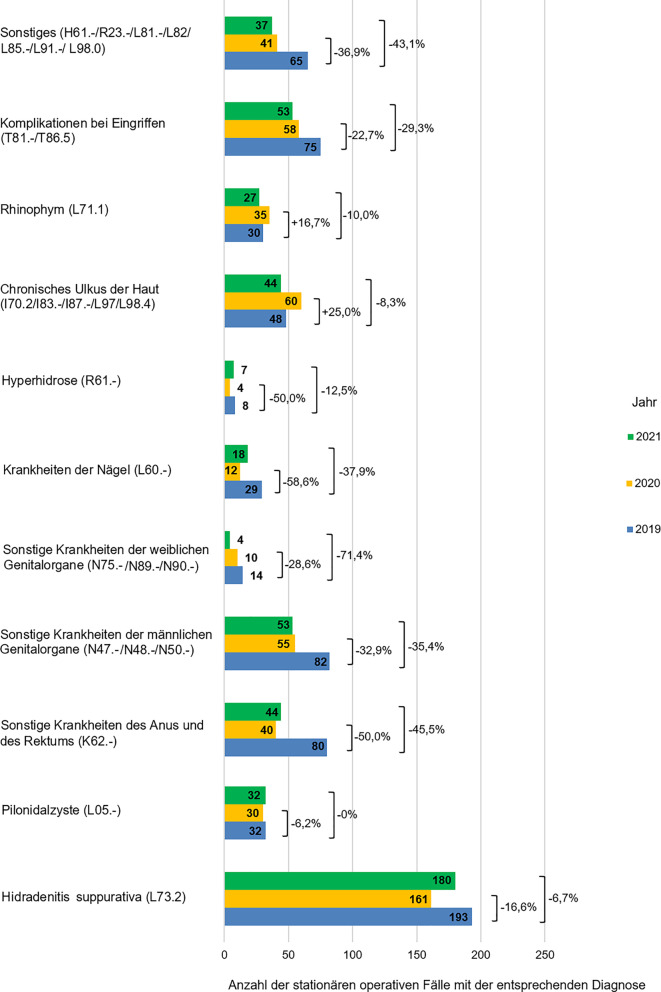
Darstellung der stationär operierten sonstigen Diagnosen in den Jahren 2019 bis 2021 mit prozentualem Rückgang der häufigsten Diagnosen in den Jahren 2020 und 2021 im Vergleich zum Jahr 2019 (als 100 % angenommen)

Insgesamt lag der Rückgang der stationären Fälle für viele weitere operativ versorgte Hauterkrankungen im Jahr 2020 zwischen 28,6 % (Sonstige Krankheiten der weiblichen Genitalorgane, ICD-Diagnosen N75.-, N79.- und N90.‑) und 58,6 % (Krankheiten der Nägel, ICD-Diagnose L60.‑). Im Jahr 2021 war die Entwicklung der stationären Fälle für diese Diagnosen inhomogen. Für einige Krankheitsbilder zeigte sich eine weitere Reduktion der stationären operativen Fälle (wie für sonstige Krankheiten der weiblichen Genitalorgane oder sonstige Krankheiten der männlichen Genitalorgane), für andere zeigte sich eine Zunahme der stationären Fälle gegenüber dem Jahr 2020, auch wenn das Niveau des Jahres 2019 noch nicht erreicht wurde (wie Hyperhidrose oder Krankheiten der Nägel). Zu beachten ist, dass die Codes A63.- und N48.- häufig synonym verwendet werden. Hier wurden Condylomata acuminata jedoch primär mit Code A63.- codiert und Code N48.- beispielsweise für Balanitis plasmacellularis Zoon oder Lichen sclerosus genutzt. (Abb. 7).

Für schwerwiegende und schmerzhafte Erkrankungen wie Hidradenitis suppurativa und Pilonidalsinus zeigte sich ein deutlich geringerer Rückgang der stationär operierten Fälle im Jahr 2020 und 2021 im Vergleich zu 2019 um jeweils 16,6 % (32 Fälle) und 6,2 % (2 Fälle) (Abb. 7).

Im Gegensatz dazu stieg im Jahr 2020 die Anzahl stationär operierter Ulcera cruris um 25 % (12 Fälle), auch wenn im Jahr 2021 ein geringer Rückgang (um 8,3 % oder 4 Fälle) beobachtet wurde. Zudem wurden im Jahr 2020 um 16,7 % (5 Fälle) mehr Rhinophyme stationär operiert als im Jahr 2019 (Abb. 7).

## Diskussion

Zum Einfluss der COVID-19-Pandemie auf die stationäre Versorgung dermatochirurgischer Patienten in Deutschland liegen bisher nur wenige veröffentlichte Daten vor. Eine retrospektive bundesweite Auswertung der stationär operierten Hauttumoren (maligne Melanome und epitheliale Tumoren („non-melanoma skin cancer“ [NMSC])) zeigte einen Rückgang der stationären Gesamtfallzahlen im Zeitraum vom 18.03.2020 bis 17.03.2021 um 14 % verglichen zum gleichen Zeitraum des Vorjahres [14]. Obwohl es sich hierbei nur um ausgewählte Diagnosen handelte, zeigen diese Ergebnisse eine Übereinstimmung mit den von uns ausgewerteten Daten von 9 Kliniken aus 4 verschiedenen Bundesländern, die einen Rückgang aller stationären operativen Fälle im Jahr 2020 um 14,5 % im Vergleich zum Jahr 2019 aufdecken. Die erwähnte Arbeit zeigte zudem, dass die Anzahl der stationär operierten Melanome im Rahmen der COVID-19-Pandemie weniger stark rückläufig war als die Anzahl der NMSC-Fälle. Unsere Daten bestätigen diese Ergebnisse. Darüber hinaus gibt unsere retrospektive Analyse interessante Einblicke in die Entwicklung der stationären Dermatochirurgie im weiteren Verlauf der Pandemie. So konnte im Jahr 2021 die Anzahl der stationär operierten Patienten nicht das Niveau vor der Pandemie erreichen und zeigte sich um 8,5 % geringer als im Jahr 2019. Dies steht im Einklang mit den Ergebnissen einer Befragung der deutschen dermatologischen Kliniken im 1. Quartal 2022. Nur knapp ein Drittel der befragten Kliniken gab an, dass die Anzahl dermatochirurgischer Fälle sich vollständig auf das Ausgangsniveau vor der Pandemie „erholt“ habe [13]. Dabei zeigen unsere Daten, dass die Entwicklung der stationären operativen Fallzahlen überdies auch abhängig von der vorliegenden Diagnose war. Die stationäre dermatochirurgische Versorgung der Melanome erreichte im Jahr 2021 beinahe das Niveau aus dem Jahr 2019, und es zeigte sich nur ein kleiner Rückgang dieser stationären Diagnose im Jahr 2021 um 1,4 % im Vergleich zum Jahr vor der COVID-19-Pandemie. Auch die Anzahl der an NMSC stationär operierten Patienten im Jahr 2021 stieg im Vergleich zum Jahr 2020 an und war nur um 5,6 % geringer als im Jahr 2019. Dies kann unter anderem durch die Verbesserung der personellen Ressourcen in dermatologischen Kliniken erklärt werden. So berichtete im Rahmen der bereits erwähnten Umfrage knapp ein Drittel der dermatologischen Kliniken, dass das ärztliche Personal der Klinik zu Beginn der Pandemie oft für andere Arbeitsgebiete abgezogen wurde. Im 1. Quartal 2022 gaben dies nur noch 6,7 % der befragten Kliniken an. Es ist aber auch denkbar, dass mit den sinkenden COVID-19-Fallzahlen und Abschluss der Impfkampagne viele Patienten, die vorher aus Sorge vor Ansteckung Arztpraxen und Krankenhäuser mieden, nun wieder vermehrt dermatologisch vorstellig wurden und entsprechend operativ versorgt werden konnten.

Die Entwicklung der stationären Versorgung von In-situ-Läsionen im Rahmen der COVID-19-Pandemie zeigt ein inhomogenes Bild. Übereinstimmend mit den Daten von Kleemann et al. zeigte sich in unserer Auswertung ein deutlicher Rückgang der stationär operierten In-situ-Malignome im 1. Jahr der COVID-19-Pandemie verglichen zum Jahr davor [14]. So war im Jahr 2020 ein Rückgang sowohl der stationär operierten In-situ-Melanome (um 18,1 %) als auch der In-situ-Karzinome (30,3 und 33,5 %, s. Abb. 3) im Vergleich zum Jahr vor der Pandemie zu verzeichnen. Allerdings zeigte sich im Jahr 2021 ein Anstieg der stationär operierten In-situ-Melanome um 17,7 % verglichen zum Jahr 2019, während für In-situ-Karzinome im Jahr 2021 ein weiterer Rückgang der Fallzahlen (um 35,2 und 59,9 %) im Vergleich zum Jahr 2019 beobachtet werden konnte. Vermutlich kann diese Tendenz nicht nur den Auswirkungen der SARS-CoV-2-Pandemie zugeschrieben werden. Vielmehr kann diskutiert werden, ob es sich um eine zusätzliche Auswirkung der seit Jahren zunehmenden Einschränkungen bei der Vergütung stationär erbrachter Leistungen handelt [18]. Die Restriktionen im Rahmen der Pandemie könnten diesen Trend zusätzlich beschleunigt haben. Bestimmte Diagnosen wie aktinische Keratosen werden jetzt vermutlich häufiger ambulant behandelt.

Seltenere Tumordiagnosen wie atypisches Fibroxanthom (ICD-Diagnose D48.‑) oder kutane Sarkome (ICD-Diagnosen C46.-, C49.-, C50.-, C75.- und C76.‑) zeigten auch im Rahmen der COVID-19-Pandemie keinen starken Rückgang der stationären operativen Fälle. Im Gegenteil kam es sowohl im Jahr 2020 als auch im Jahr 2021 zu einer Zunahme der stationär operierten atypischen Fibroxanthome um jeweils 9,3 und 16,7 % verglichen mit dem Jahr 2019. Obwohl es im Jahr 2020 zu einem Rückgang der stationär operierten kutanen Sarkome um 8,3 % im Vergleich zum Jahr 2019 kam, stieg die Fallzahl der Sarkomdiagnosen im Jahr 2021 um 6,6 %. Ein möglicher Grund dafür ist, dass es sich bei diesen Tumorentitäten oft um schnell wachsende Läsionen handelt, die auch während der COVID-19-Pandemie von Patienten nicht ignoriert werden konnten bzw. deren Behandlung nicht aufgeschoben werden konnte. Ein anderer Grund dafür ist die allgemeine Zunahme dieser Diagnosen in der Allgemeinbevölkerung, wie eine jüngst veröffentliche Studie aus Deutschland zeigen konnte [19]. Die stationäre operative Versorgung der kutanen Lymphome (ICD-Diagnosen C82.-, C83.-, C84.-, C85.- und C86.‑) und der Tumormetastasen (sekundäre bösartige Neubildungen, ICD-Diagnosen C77.-, C79.-, C80.- und C97.‑) ging im Jahr 2020 ähnlich wie bei vielen anderen Diagnosen (um jeweils 33,7 und 16,6 % verglichen zum Jahr 2019) zurück. Im Jahr 2021 kam es zu einer erneuten Zunahme, die aber noch nicht das Niveau vor der Pandemie erreicht hat (Abb. 4). Vermutlich verhinderten weiterhin bestehende Einschränkungen der personellen Ressourcen und Bettenkapazitäten im Jahr 2021 eine stationäre dermatochirurgische Versorgung auf dem Niveau des Jahres 2019. Auch im 1. Quartal 2022 berichteten 31,1 % der an einer Befragung teilnehmenden dermatologischen Kliniken immer wieder, stationäre Betten zugunsten der COVID-nahen Fachrichtungen (z. B. COVID-Stationen) abgeben zu müssen [13]. Als Konsequenz für diese nur langsame Rückkehr zur Normalität konnte in deutschen dermatologischen Kliniken eine Zunahme der besonders dicken (> 6 mm) Plattenepithelkarzinome sowie der positiven Schildwächterlymphknoten bei malignen Melanomen sowohl im Jahr 2020 als auch im Jahr 2021 beobachtet werden [16, 17].

Gutartige Läsionen der Haut und Subkutis wurden im Jahr 2020 deutlich seltener stationär operiert als im Jahr vor der COVID-19-Pandemie (Abb. 5). Dazu zählen folgende Diagnosen: Melanozytennävus (ICD-Diagnose D22.‑), gutartige Neubildungen des Bindegewebes (ICD-Diagnosen D17.-, D18.-, D21.‑) und follikuläre Zysten der Haut und Unterhaut (ICD-Diagnose L72.‑). Im Jahr 2021 kam es zu einer langsamen Zunahme der stationären Fälle mit der Hauptdiagnose Melanozytennävus und gutartige Neubildungen des Bindegewebes im Vergleich zum Vorjahr, aber das Niveau des Jahres 2019 wurde nicht erreicht. Interessanterweise zeigte sich eine starke Zunahme der stationär operierten Patienten mit der Diagnose follikuläre Zysten der Haut und Unterhaut. Die Anzahl dieser stationären Fälle überstieg die Fälle des Jahres 2019 im Jahr 2021 um 14,3 %. Auch die Anzahl der stationär operierten Fälle mit den Diagnosen gutartige Neubildungen der Haut (ICD-Diagnosen D23.-, D28.-, D29.-, D36.‑) und sonstige angeborene Fehlbildungen der Haut und Gefäße (ICD-Diagnosen Q17.-, Q27.-, Q82.-, Q85.‑) stieg sowohl im Jahr 2020 als auch im Jahr 2021 im Vergleich zum Jahr 2019 an. Der Grund dieser inhomogenen Entwicklung der unterschiedlichen gutartigen Diagnosen ist bislang unklar. Möglicherweise spielt auch das Alter der betroffenen Patienten eine Rolle. In der Regel werden mit der Diagnose Melanozytennävus im stationären Rahmen Kinder operiert [20, 21]. Unsere Auswertung zeigte jedoch einen statistisch signifikanten Rückgang der stationär operierten Kinder in den beiden Pandemiejahren im Vergleich zum Jahr 2019. Diese Tendenz zeigt unter anderem, dass viele Eltern aufgrund möglicher schwerer COVID-19-Verläufe im Kindesalter [22] nicht bereit waren, ihre Kinder einer vermuteten Gefahr der Ansteckung im Krankenhaus auszusetzen. Zudem kann angenommen werden, dass bei weiterhin eingeschränkten Personal- und Bettenressourcen elektive Eingriffe bei gutartigen Hautveränderungen seitens der dermatologischen Kliniken zurückgestellt wurden. Ausgedehnte dermatochirurgische Eingriffe, die einen stationären Aufenthalt erforderlich machen, werden im Kindesalter in den meisten Kliniken in Vollnarkose durchgeführt. Die Allokation von Anästhesisten zur intensivmedizinischen Versorgung von COVID-19-Patienten könnte ebenfalls zum relevanten Rückgang der operierten kongenitalen Nävi geführt haben. Bei erwachsenen Patienten erfolgt die Durchführung überwiegend in Infiltrationslokalanästhesie oder Tumeszenzlokalanästhesie, was einen immensen Vorteil gegenüber anderen chirurgischen Disziplinen darstellte, da damit keine Anästhesiekapazitäten gebunden wurden.

Auch infektiologische Diagnosen wie Viruswarzen (ICD-Diagnose B07.‑), Hautabszesse und Furunkel (ICD-Diagnosen L02.-, L03.‑) wurden im Jahr 2020 seltener stationär operiert als im Jahr 2019, und im Jahr 2021 ging die stationäre Anzahl dieser Diagnosen noch weiter zurück. Allerdings nahm die Anzahl der stationär operierten Patienten mit anogenitalen (venerischen) Warzen (ICD-Diagnose A63.‑) im Jahr 2020 im Vergleich zum Jahr 2019 zu. Eine mögliche Erklärung für dieses Phänomen ist, dass es sich bei Patienten mit dieser Diagnose meistens um junge Erwachsene handelt [23], die grundsätzlich weniger schwere COVID-19-Verläufe im Vergleich zu anderen Altersgruppen zu befürchten hatten.

Unter anderen gutartigen Diagnosen sollen die Hidradenitis suppurativa (ICD-Diagnose L73.2) und das Ulcus cruris (chronisches Ulkus der Haut, ICD-Diagnosen I70.2, I83.-, L97, L98.4) aufgrund ihrer Häufigkeit hervorgehoben werden. Bei der Hidradenitis suppurativa (HS) handelt es sich um eine schmerzhafte Erkrankung, die mit starker Beeinträchtigung der Lebensqualität einhergeht [24]. Daher ist es nicht verwunderlich, dass es im Jahr 2020 zu einem nur moderaten Rückgang (um 16,6 %) der stationär operierten Fälle mit dieser Diagnose verglichen zum Jahr 2019 kam und im Jahr 2021 dieser Rückgang (um nun 6,7 %) fast komplett aufgeholt werden konnte. Obwohl die Erkrankung selbst nicht zu einer gesteigerten Anfälligkeit oder schwereren Krankheitsverläufen im Hinblick auf COVID-19-Infektionen prädisponiert [25], war die Versorgung der HS-Patienten während der Pandemie beeinträchtigt [26]. Eine Patientenumfrage aus Deutschland zeigte, dass die stationären Aufenthalte der betroffenen Patienten in 8 % der Fälle aufgrund der Pandemie verschoben und in 2 % der Fälle sogar abgesagt wurden. Viele Patienten berichteten zudem, aus Angst vor Ansteckung mit dem SARS-CoV-2-Virus generell Arztbesuche vermieden oder reduziert zu haben [26]. Unsere Daten spiegeln diese Tendenz wider und zeigen, dass die operative Versorgung der HS eine wichtige Therapieoption darstellt, die im Jahr 2021 beinahe das Niveau vor der COVID-19-Pandemie erreicht hat (Abb. 7).

Die operative Versorgung der Patienten mit Ulcus cruris nahm dagegen im 1. Pandemiejahr um 25,0 % im Vergleich zum Jahr 2019 zu. Zwar ergab eine Patientenumfrage aus Deutschland Hinweise auf einen eingeschränkten Zugang der Ulkuspatienten zur ärztlichen Versorgung in den Kliniken, dies galt jedoch nicht für Praxen oder Wundzentren. Diese Daten sind in ihrer Aussagekraft eingeschränkt, da es sich hierbei um eine kleine Stichprobe aus 63 Patienten handelte, welche die Umfrage per E‑Mail erhalten haben [27]. Somit konnten vulnerable Patientengruppen wie sehr alte oder demente Patienten nicht erfasst werden. Eine Studie aus Großbritannien analysierte retrospektiv knapp 2000 Patienten mit chronisch venösen Ulzera in den Jahren 2019, 2020 und 2021 [28]. Hierbei zeigte sich, dass diese Patienten während der COVID-19-Pandemie einen deutlich eingeschränkteren Zugang zum Gesundheitswesen als im Jahr 2019 hatten, die Verbandswechsel seltener erfolgten und die Ulzera schlechter geheilt sind. Unsere Auswertung erfasste nur Patienten mit chronischen Ulzera, die eine chirurgische Wunddefektdeckung (Spalthauttransplantation) erhalten haben. Patienten, die im Rahmen des stationären Aufenthaltes nur eine chirurgische Wundreinigung (Débridement) erhalten haben, wurden aus der Analyse ausgeschlossen. Somit zeigte sich im 1. Pandemiejahr eine Zunahme der Spalthauttransplantationen bei Patienten mit Ulcus cruris. Möglicherweise führte eine reduzierte ambulante Versorgung der chronischen Wunden zum verstärkten Wunsch der Patienten, einen definitiven Wundverschluss zu erreichen, und somit zu einer höheren Akzeptanz dermatochirurgischer Methoden. Im 2. Pandemiejahr zeigte sich ein leichter Rückgang der operativ versorgten Patienten mit Ulcera cruris, was möglicherweise durch eine Verbesserung der ambulanten Betreuung nach Einführung der COVID-19-Vakzine erklärt werden kann.

Die Limitation unserer Arbeit besteht möglicherweise darin, dass nur 9 Hautkliniken aus 4 Bundesländern an der Auswertung teilgenommen haben. Die Ergebnisse unserer Arbeit zur dermatochirurgischen Versorgung von Melanomen und NMSC korrelieren hierbei mit den Daten einer bundesweiten Studie [14]. Dies lässt vermuten, dass es sich bei den von uns erhobenen Daten aus den 9 beteiligten Kliniken um eine repräsentative Stichprobe handelt und auch die weiteren von uns betrachteten Diagnosen repräsentative Daten darstellen. Grundsätzlich wären eine längere Verlaufsbeurteilung und Betrachtung der Entwicklung der stationären dermatochirurgischen Fälle in den Jahren 2022 und 2023 von Interesse, auch wenn im Jahr 2023 nach Beendigung der COVID-19-Pandemie eine vollständige Erholung der stationären Dermatochirurgie zu erwarten ist.

Zusammenfassend liefert unsere Arbeit eine umfassende, diagnosebezogene Übersicht über die Entwicklungen der stationären dermatochirurgischen Versorgung in Deutschland. Insbesondere im 1. Pandemiejahr war die stationäre Dermatochirurgie stark beeinträchtigt. Unsere Daten zeigen aber auch, dass trotz eingeschränkter personeller Ressourcen und reduzierter Bettenzahl die operative Versorgung wichtiger Krankheitsbilder wie bösartiger Tumoren der Haut (Melanom, NMSC) und des Bindegewebes (Sarkome) nur mit einer verhältnismäßig geringen Verzögerung gewährleistet war und im 2. Pandemiejahr beinahe unverändert zum Jahr 2019 erfolgte – im Gegensatz zur Versorgung vieler gutartiger Erkrankungen. Unsere Studie zeigt, wie sich die stationäre Versorgung des gesamten dermatochirurgischen Erkrankungsspektrums im Rahmen der COVID-19-Pandemie entwickelte, auch wenn weitere Einflussfaktoren bezüglich der stationären Versorgung bestimmter gutartiger Diagnosen wie beispielsweise wirtschaftlicher Druck seitens der Krankenkassen zusätzlich vermutet werden können. Insgesamt zeigte sich die stationäre Dermatochirurgie jedoch auch im 2. Jahr der COVID-19-Pandemie beeinträchtigt, was sich in einem Rückgang der stationären operativen Fälle um 8,5 % im Vergleich zum Jahr 2019 äußerte. Dennoch konnte im Jahr 2021 eine erneute Zunahme der stationären dermatochirurgischen Fälle im Vergleich zum 1. Pandemiejahr beobachtet werden. Ein wesentlicher Vorteil der Dermatochirurgie gegenüber anderen chirurgischen Fächern, der im Rahmen der COVID-19-Pandemie nicht außer Acht gelassen werden darf, beruht zum großen Teil auf dem Einsatz von Infiltrationslokalanästhesie und Tumeszenzlokalanästhesie, was sogar zur Adaptierung durch andere Fachrichtungen geführt hat [29]. Dennoch kann die oben gezeigte positive Tendenz als ein Hinweis für einen weiterhin bestehenden starken Bedarf an stationärer dermatochirurgischer Versorgung interpretiert werden, der insbesondere in Anbetracht der kontinuierlich steigenden Inzidenzzahlen der NMSC in Deutschland [30] und aufgrund des zunehmenden Patientenalters sowie der oft erheblichen Tumorgröße und -anzahl ambulant nicht aufgefangen werden kann. Mögliche Langzeitfolgen der COVID-19-Pandemie auf die stationäre Versorgung dermatochirurgischer Patienten bleiben abzuwarten. Berufliche Neuorientierung von Krankenhauspersonal, mangelnder ärztlicher und pflegerischer Nachwuchs und steigender Druck durch Ambulantisierung und Bettenkürzungen sind nur einige Beispiele. Eine langfristige Verlaufsbeurteilung der Entwicklung der stationären dermatochirurgischen Fälle ist daher in Zukunft von großem Interesse.

## Supplementary Information

Online-Tabellen 1, 2 und 3: Liste der an der Datenauswertung teilnehmenden Zentren und Darstellung aller nach ICD-10 codierten dermatochirurgischen stationären Hauptdiagnosen in den teilnehmenden Kliniken.

## Data Availability

Die Daten können auf angemessene Anfrage und bei Nennung der Gründe für die Notwendigkeit der Dateneinsicht zur Verfügung gestellt werden. Sie können dann bei der Korrespondenzautorin angefragt werden.
