# Supplementary material for: Effects of the COVID-19 pandemic on inpatient dermatosurgery in Germany: Retrospective evaluation of the surgical cases from nine dermatology clinics
Source: Dermatologie (Heidelb). 2024 Oct 10;75(11):864–77. [Article in German] doi: 10.1007/s00105-024-05417-5 (PMC11538252; doi:10.1007/s00105-024-05417-5)
Supplement: Supplementary file 1 — Online-Tabellen 1, 2 und 3: Liste der an der Datenauswertung teilnehmenden Zentren und Darstellung aller nach ICD-10 codierten dermatochirurgischen stationären Hauptdiagnosen in den teilnehmenden Kliniken. [file 105_2024_5417_MOESM1_ESM.pdf]

Supplementary Information: Online-Tabellen 1, 2 und 3

Online-Tabelle 1: Liste der an der Datenauswertung teilnehmenden Zentren (alphabetisch sortiert).

Teilnehmende Klinik	Affiliation mit einer Universität	Bundesland
Klinik für Dermatologie und Allergologie, Universitätsklinikum der RWTH Aachen, Aachen	Universitätsklinik	Nordrhein-Westfalen
Zentrum für Hauterkankungen, Universitätsklinikum Bonn, Bonn	Universitätsklinik	Nordrhein-Westfalen
Klinik für Dermatologie und Venerologie, HELIOS Klinikum Krefeld, Krefeld	Lehrkrankenhaus einer Universitätsklinik	Nordrhein-Westfalen
Hautklinik, Klinikum Ludwigshafen am Rhein, Ludwigshafen	Lehrkrankenhaus einer Universitätsklinik	Rheinland-Pfalz
Abteilung für Dermatochirurgie und Dermatologie, Artemed Fachklinik München, München	Keine Affiliation	Bayern
Klinik und Poliklinik für Dermatologie und Allergologie, LMU München, München	Universitätsklinik	Bayern
Klinik für Dermatologie, Venerologie und Allergologie, HELIOS St. Elisabeth Klinik Oberhausen, Oberhausen	Lehrkrankenhaus einer Universitätsklinik	Nordrhein-Westfalen
Hautklinik, Universitätsklinikum Tübingen, Tübingen	Universitätsklinik	Baden-Württemberg
Zentrum für Dermatologie, Allergologie und Dermatochirurgie, HELIOS Universitätsklinikum Wuppertal	Universitätsklinik	Nordrhein-Westfalen

Online-Tabelle 2: Darstellung aller nach ICD-10 codierten dermatochirurgischen stationären Hauptdiagnosen in den teilnehmenden Kliniken, alphabetisch sortiert.

**ICD-10-Diagnoseschlüssel**	**Aufschlüsselung der Diagnose**
A63.-	Anogenitale (venerische) Warzen
B07.-	Viruswarzen
B08.-	Molluscum contagiosum
C00.-	Bösartige Neubildung der Lippe
C02.-	Bösartige Neubildung der Zunge
C03.-	Bösartige Neubildung vom Unterkieferzahnfleisch

C21.-	Bösartige Neubildung des Anus
C43.-	Bösartiges Melanom der Haut
C44.-	Sonstige bösartige Neubildungen der Haut (wie Basalzellkarzinom, Plattenepithelkarzinom)
C46.-	Kaposi-Sarkom
C49.-	Bösartige Neubildung sonstigen Bindegewebes und anderer Weichteilgewebe (wie pleomorphes dermales Sarkom)
C50.-	Bösartige Neubildung der Mamma
C51.-	Bösartige Neubildung der Vulva
C60.-	Bösartige Neubildung des Penis
C63.-	Bösartige Neubildung sonstiger und nicht näher bezeichneter männlicher Genitalorgane
C75.-	Bösartige Neubildung sonstiger endokriner Drüsen und verwandter Strukturen
C76.-	Bösartige Neubildung sonstiger und ungenau bezeichneter Lokalisationen
C77.-	Sekundäre und nicht näher bezeichnete bösartige Neubildung der Lymphknoten
C79.-	Sekundäre bösartige Neubildung an sonstigen und nicht näher bezeichneten Lokalisationen
C80.-	Bösartige Neubildung ohne Angabe der Lokalisation
C82.-	Follikuläres Lymphom
C83.-	Nicht follikuläres Lymphom
C84.-	Reifzellige T/NK-Zell-Lymphome
C85.-	Sonstige und nicht näher bezeichnete Typen des Non-Hodgkin-Lymphoms
C86.-	Weitere spezifizierte T/NK-Zell-Lymphome
C97.-	Bösartige Neubildungen als Primärtumoren an mehreren Lokalisationen
D03.-	Melanoma in situ
D04.-	Carcinoma in situ der Haut (beispielsweise Morbus Bowen)
D07.-	Carcinoma in situ der Genitalorgane
D17.-	Gutartige Neubildung des Fettgewebes
D18.-	Hämangiom und Lymphangiom

D21.-	Sonstige gutartige Neubildungen des Bindegewebes und anderer Weichteilgewebe
D22.-	Melanozytennävus
D23.-	Sonstige gutartige Neubildung der Haut
D28.-	Gutartige Neubildungen der weiblichen Genitalorgane
D29.-	Gutartige Neubildungen der männlichen Genitalorgane
D36.-	Gutartige Neubildung an sonstigen und nicht näher bezeichneten Lokalisationen
D48.-	Neubildung unsicheren oder unbekannten Verhaltens an sonstigen und nicht näher bezeichneten Lokalisationen (beispielsweise auch atypisches Fibroxanthom)
H61.-	Sonstige Krankheiten des äußeren Ohres
I70.2	Atherosklerose der Extremitätenarterien
I83.-	Varizen der unteren Extremitäten
I87.-	Sonstige Venenkrankheiten
K62.-	Sonstige Krankheiten des Anus und des Rektums
L02.-	Hautabszess, Furunkel und Karbunkel
L03.-	Phlegmone
L05.-	Pilonidalzyste
L57.-	Hautveränderungen durch chronische Exposition gegenüber nichtionisierender Strahlung (aktinische Keratosen)
L60.-	Krankheiten der Nägel
L71.1	Rhinophym
L72.-	Follikuläre Zysten der Haut und der Unterhaut (beispielsweise Atherom oder Trichilemmalzyste)
L73.2	Hidradenitis suppurativa
L81.-	Sonstige Störungen der Hautpigmentierung
L82	Seborrhoische Keratose
L85.-	Sonstige Epidermisverdickung (inklusive Cornu cutaneum)
L91.-	Hypertrophe Hautkrankheiten (inklusive hypertrophe Narben)
L97	Ulcus cruris, anderenorts nicht klassifiziert
L98.4	Chronisches Ulkus der Haut, anderenorts nicht klassifiziert
L98.0	Granuloma pediculatum (Granuloma pyogenicum)

N47.-	Vorhauthypertrophie, Phimose und Paraphimose
N48.-	Sonstige Krankheiten des Penis
N50.-	Sonstige Krankheiten der männlichen Genitalorgane
N75.-	Krankheiten der Bartholini-Drüsen
N89.-	sonstige nichtentzündliche Krankheiten der Vagina
N90.-	sonstige nichtentzündlichen Krankheiten der Vulva und des Perineums
Q17.-	Sonstige angeborene Fehlbildungen des Ohres
Q27.-	Sonstige angeborene Fehlbildungen des peripheren Gefäßsystems
Q82.-	Sonstige angeborene Fehlbildungen der Haut
Q85.-	Phakomatosen, anderenorts nicht klassifiziert
R23.-	Sonstige Hautveränderungen
R61.-	Hyperhidrose
T81.-	Komplikationen bei Eingriffen, anderenorts nicht klassifiziert
T86.5	Versagen und Abstoßung eines Hauttransplantates

Online-Tabelle 3: Darstellung der ausgewerteten Diagnosegruppen. Um die Datenauswertung übersichtlicher zu gestalten, wurden pathophysiologisch ähnliche Diagnosen oder Diagnosen an gleichen Lokalisationen zu Diagnosegruppen zusammengefasst. Die letzte Diagnosegruppe („Sonstiges“) beinhaltet alle seltenen stationären Diagnosen, die thematisch keiner anderen Gruppe zugeordnet werden konnten.

**Zusammengefasste ICD-10-Diagnoseschlüssel**	**Resultierende Diagnosegruppen**
C00.-/C02.-/C03.-	Bösartige Neubildungen der Lippen, Zunge oder Gingiva
C46.-/C49.-/C50.-/ C75.-/C76.-	Bösartige Neubildung des Bindegewebes (inklusive Kaposi-Sarkom und pleomorphes dermales Sarkom) jeder Lokalisation (außer Genitoanalbereichs)
C21.-/C51.-/C60.-/C63.-	Bösartige Neubildung des Genitoanalbereichs
C77.-/C79.-/C80.-/C97.-	Sekundäre bösartige Neubildung jeder Lokalisation
C82.-/C83.-/C84.-/C85.-/C86.-	(Kutane) Lymphome
D04.-/D07.-	Carcinoma in situ der Haut und Genitalorgane
D17.-/D18.-/D21.-	Gutartige Neubildung des Bindegewebes

D23.-/D28.-/D29.-/D36.-	Gutartige Neubildungen der Haut und sonstigen Lokalisationen
I70.2/I83.-/I87.-/L97/L98.4	Chronisches Ulkus der Haut (inklusive Ulcus cruris) unabhängig von der Genese
L02.-/L03.-	Hautabszess, Furunkel, Karbunkel oder Phlegmone
N47.-/N48.-/N50.-	Sonstige Krankheiten der männlichen Genitalorgane
N75.-/N89.-/N90.-	Sonstige Krankheiten der weiblichen Genitalorgane
Q17.-/Q27.-/Q82.-/Q85.-	Sonstige angeborene Fehlbildungen der Haut und Gefäße
T81.-/T86.5	Komplikationen bei Eingriffen
H61.-/R23.-/L81.-/L82/ L85.-/L91.-/ L98.0	Sonstiges
